# Once Dishonest, Always Dishonest? The Impact of Perceived Pervasiveness of Moral Evaluations of the Self on Motivation to Restore a Moral Reputation

**DOI:** 10.3389/fpsyg.2016.00586

**Published:** 2016-04-26

**Authors:** Stefano Pagliaro, Naomi Ellemers, Manuela Barreto, Cecilia Di Cesare

**Affiliations:** ^1^Laboratory of Social Psychology, Department of Neuroscience, Imaging and Clinical Sciences, University of Chieti-PescaraChieti, Italy; ^2^Laboratory of Social Health and Organizational Psychology, Institute of Psychology, Faculty of Social Sciences, Utrecht UniversityUtrecht, Netherlands; ^3^Department of Psychology, University of ExeterExeter, UK

**Keywords:** morality, competence, sociability, pervasiveness, reparation

## Abstract

Four studies specify how moral evaluations of the self regulate behavior aimed at restoring a moral reputation. We propose that people care about evaluations of themselves as moral or immoral because these are perceived as more consequential than other types of information. Therefore people are more inclined to restore their image after being negatively evaluated in terms of morality rather than competence. Studies 1 and 2 revealed that moral information was perceived as having a more enduring impact on one’s reputation, and was more strongly related to anticipate intra-group respect and self-views, than competence and sociability information. This perceived pervasiveness of moral (vs. competence) evaluations mediated intentions to justify and explain one’s behavior (Study 3). Study 4 finally showed that being seen as lacking in morality elicited threat and coping responses, which induced subsequent tendencies to repair one’s moral reputation.

## Introduction

Moral judgments distinguish ‘right’ from ‘wrong.’ They indicate standards of human virtue, and serve as a guideline for individual behavior (Beauchamp, 2001). Nevertheless, we encounter a steady stream of cases in which scientists, sportsmen, public administrators, or bankers, lie, cheat, steal, or demonstrate other types of behavior we tend to see as immoral. This raises the question whether people actually *care* about whether or not their behavior is seen by others as moral. Indeed, people often seem to focus primarily on demonstrating their competence and achieving (financial) success—even if this means generally behaving in ways that tend to be seen as immoral. In this paper, we therefore examine how people respond to the way their behavior is evaluated by others, systematically comparing concerns raised due to moral evaluations of the self with concerns others may have about one’s competence.

Prior research has revealed that moral evaluations are central to people’s judgments of *other* individuals or groups (Pagliaro, 2012; Brambilla and Leach, 2014; Goodwin et al., 2014). We complement these insights by investigating the centrality of moral evaluations of the *self* and how this affects impression management. Research shows that individuals regard negative communion based judgments of the self as more threatening for their reputation than negative agency judgments, and this motivates them to restore a positive reputation in the eyes of significant others (Ybarra et al., 2012). Nevertheless, the underlying mechanisms are still unknown, as is the role of the more specific moral (vs. the more generic warmth) dimension in this process (Leach et al., 2007). At the same time, Leach et al. (2007) have demonstrated that – within the more generic warmth or communion dimension – moral characteristics (such as honesty and trustworthiness) are distinct from sociability characteristics (such as kindness and likeability). We further address this distinction, and propose that moral information is seen to pervade more aspects of one’s reputation and to have a more enduring impact on the overall impression others form about the self—i.e., it is more *pervasive—*than competence or sociability information. Thus, negative judgments of the self that bear on morality (vs. competence or sociability) are predicted to be more aversive and stressful and thereby more likely to motivate people to repair their reputation.

### The Relevance of Morality in Impression Formation

A distinction is typically made between judgments on task-relevant dimensions (agency or ‘competence’) and judgments of relational characteristics (benevolence or ‘warmth’). This work has established that people give priority to establishing the relational implications of social information—to assess whether others are likely to be helpful or harmful to the self—before they attend to information indicating their relative competence (e.g., Martijn et al., 1992; De Bruin and Van Lange, 2000; Wojciszke, 2005; Cuddy et al., 2008). This is evident from self-reports (Cuddy et al., 2008), response latencies (Willis and Todorov, 2006), and neuro-imaging data (Winston et al., 2002), as well as from the ability to correctly remember social information (Van Leeuwen et al., 2012). However, to assess judgments of benevolence researchers have tended to conflate information pertaining to *sociability* (being friendly or good-natured) with information conveying *morality* (being honest or sincere).

Recently, it has become clear that information regarding *morality—*rather than sociability—is the key ingredient determining responses to relational information about individuals and groups. Moral information is more decisive than information about sociability or competence in determining the overall impression people form of other individuals (Brambilla et al., 2011) and groups (Brambilla et al., 2012). It also is the primary determinant of the likelihood that people will approach and help others, instead of avoiding them (Pagliaro et al., 2013; Iachini et al., 2015). Thus, research has established that moral information dominates initial impression formation of, and behavioral tendencies toward, other individuals and groups (Leach et al., 2014).

The conclusion that moral information seems to have a special status when forming impressions of others raises the question whether moral information might have a similar impact when relating to the *self*. Our aim in this paper is to extend existing insights on moral impression formation, by examining the role of moral information in impression *management* (see also Ybarra et al., 2012), and addressing the social *function* of moral judgments as a way to coordinate behavior of individuals living together in groups (Ellemers and van den Bos, 2012). Prior research has established the emergence of threat and self-defensive responses when others seem superior to the self in the moral domain (Monin, 2007; Jordan and Monin, 2008; Täuber et al., 2014). Such defensive responses have also been documented when the morality of one’s group is called into question (Gausel et al., 2012; Täuber and van Zomeren, 2012). We go one step further by examining the origins and nature of the threat implied in seeming immoral. In doing this, we build on prior work (see also Gausel and Leach, 2011) suggesting that moral judgments are used to decide about *social inclusion* and that people adhere to shared moral standards as a way to secure acceptance in a group (Ellemers, 2012; Ellemers et al., 2013).

Prior work has shown that people’s moral *self-views* may affect their behavior. For instance, research on moral licensing has shown that after having affirmed their moral self-views, people are less vigilant to guard against acting immorally (e.g., Merritt et al., 2010). The reverse may also be true: after being prompted to recall one’s immoral behavior, people show a reduced tendency to cheat, allegedly as a way to compensate for prior failures, and to re-establish their moral self-view (Jordan et al., 2011). In addition to such (positive and negative) moral compensation effects, moral consistency effects have also been established. This term is used to describe the tendency of individuals to behave in ways that affirm and communicate their moral self-views (Conway and Peetz, 2012). A recent review of this work revealed that there are different aspects of the *behavior* that is considered, that may determine which of these effects is most likely to be observed (e.g., the construal level; the abstractness vs. concreteness of the behavior in question; Mullen and Monin, 2016). We extend these insights by going beyond people’s self-views, and explicitly address ‘meta-perceptions.’ That is, we assess how people *think* that *others* will perceive them, based on moral information about the self, and how this affects their behavioral intentions. Previous work suggests that – in addition to self-views of being a moral person – concerns about *other people’s views* of the moral self, may influence the extent to which individuals endorse moral-based norms and that this stems from *a desire to be respected and included by relevant others* (Pagliaro et al., 2011). We build on this initial work by further examining how the perceived image in the eyes of others impacts on the motivation to restore one’s moral reputation and relates to assessments of moral evaluations as more enduring and pervasive than evaluations about sociability or competence.

### Morality as a Source of Respect

Moral judgments can be used to define what is considered ‘good’ or ‘bad’ (Beauchamp, 2001; Brandt and Reyna, 2011). Such standards tend to be shared in communities of people living together (Haidt and Kesebir, 2010; Giner-Sorolla, 2012), as individual moral judgments are shaped by what is considered moral by others in one’s social group (Greenwood, 2011). Because moral standards indicate what behavior is expected of a ‘good’ and ‘proper’ group member, they facilitate behavioral coordination in groups (Ellemers et al., 2013). Indeed, social exclusion is seen as the ultimate sanction for failure to comply with moral group norms (Turiel, 2006; Tooby and Cosmides, 2010). Thus, there is considerable agreement that moral judgments are important because these are associated with patterns of intragroup evaluation that drive individuals to regulate their behavior in social and group contexts (Haidt and Kesebir, 2010; Rai and Fiske, 2011). To do justice to this conception of morality, we consider people’s concerns about their moral image in relation to the implications this has for the *respect they expect to receive from self-relevant others*.

In many situations, people do not think of themselves and others as separate individuals, but as members of a particular group (Tajfel, 1978; Tajfel and Turner, 1979). As a result, people’s sense of self-worth is strongly determined by whether others are willing to include and acknowledge them as ‘good’ group members. This can be achieved by acting in ways the group defines as ‘good’ and moral (Ellemers et al., 2013). While most of the work in this tradition has examined how the status of the group reflects upon individual group members, the group-value model (Lind and Tyler, 1988; Tyler and Lind, 1992) has argued for the importance of considering whether and how individuals feel they are valued *within* the group. Accordingly, it has been established that those who feel respected by others in the group report more commitment, and are more engaged with important group goals, compared to those who do not feel respected by fellow ingroup members (Tyler and Blader, 2000). When respect from other group members is not forthcoming, this produces anxiety and distress (Eisenberger et al., 2003).

Accordingly, the desire to secure respect from others in the group has been established as a fundamental motivational force. For instance, prior research revealed that individuals tend to display behaviors that indicate their deservingness of group membership, and engage in efforts that attest to their loyalty to the group (De Cremer, 2002; Jetten et al., 2002; Ellemers and Jetten, 2013). This tendency to contribute to group goals is even more pronounced when respect from other ingroup members is not forthcoming (Sleebos et al., 2006a,b; see also Huo et al., 1996; Tafarodi and Milne, 2002).

While some approaches argue for a generic ‘need to belong’ (Baumeister and Leary, 1995), our social identity analysis specifies that the importance of feeling respected depends on whether or not the *source* of respect is seen as relevant for the social self, as well as whether or not the *dimension* on which group members are evaluated is central to the group’s values (see also Spears et al., 2005). For instance, when exposed to different types of normative expectations of others, individuals were more inclined to adapt their behavior when they anticipated this to have implications for their moral image in the group rather than their competent image (Ellemers et al., 2008; Pagliaro et al., 2011).

In a prior study, Ybarra et al. (2012) compared the impact of different types of evaluations on the self. Participants were asked to imagine being the target of someone else’s suspicions and accusations, which either referred to their task abilities (‘agency,’ i.e., failing an examination), or to their honesty (‘communion,’ i.e., cheating on an examination). Participants reported being more concerned with their social acceptance and reputation when they had been asked to imagine their honesty was called into question than when their competence was doubted. However, this research did not address the question *why* moral evaluations raised such concerns, or how this predicts further attempts at reputation management. These are the aims of the present research. Prior studies have revealed that people are more inclined to see moral failures as revealing people’s ‘true character’ than competence failures (Reeder and Spores, 1983; Skowronski and Carlston, 1987; Goodwin et al., 2014). Additionally, actions that harm other people are more likely to be seen as intentional as actions that help them (Guglielmo and Malle, 2010). We propose that the special role played by moral self-evaluations stems from the fact that they are perceived as more enduring or pervasive, and therefore also as more threatening, than competence or sociability evaluations. This, in turn, motivates individuals to restore their reputation more strongly when their morality is questioned than when their sociability or competences are doubted.

### The Present Research

We argue that moral evaluations of the self are seen as pervading more aspects of, and having a more enduring impact on, one’s image in the eyes of others (i.e., as more all-encompassing and pervasive) than evaluations pertaining to alternative evaluative domains such as competence or sociability. This is expected to affect the extent to which people care about the judgments of others, the threat they experience as a result of such evaluations, and the likelihood that they are willing to make an effort to repair their image.

We set out first to assess whether moral evaluations of the self are indeed seen as more pervasive and consequential for one’s image over time than competence and sociability evaluations (*Hypothesis 1*). This was tested in Studies 1, 2, and 3 with different experimental designs and outcome measures. Second, in Studies 3 and 4 we examined whether the motivation to restore one’s image in the eyes of others is more pronounced after receiving a negative moral evaluation than after a negative competence evaluation (*Hypothesis 2*). Third, in Studies 3 and 4 we aimed to test whether the motivation to restore one’s image is associated with the desire to secure intragroup respect and inclusion, rather than reflecting a more generic concern with moral judgments (*Hypothesis 3*).

## Study 1

Study 1 aimed to show that morality judgments are perceived as having a more enduring impact on one’s image than competence evaluations. We also aimed to examine whether a negative evaluation from other ingroup members is more likely to undermine anticipated levels of intragroup respect when it pertains to morality rather than competence.

### Method

#### Participants

A total of 126 undergraduates took part in this study (82 women; mean age = 20.59; *SD* = 3.46). Participants were recruited during a Psychology class and asked to anonymously complete a paper-and-pencil questionnaire. Each session lasted approximately half an hour, after which participants were thanked and fully debriefed.

#### Procedure

Participants rated to what extent six different characteristics would pervade the resulting image of a person over time (Likert scale from 1 = *Not at all*; 9 = *Very much*). These descriptors were adapted from the scale developed by Leach et al. (2007), and referred to moral (*honest, sincere, trustworthy*; α = 0.63) and competence judgments (*competent, intelligent, skillful*; α = 0.72). A Principal Components Analysis (PCA) with Direct Oblimin rotation accounted for 61.70% of the variance in the individual items, and confirmed that the six items fell into two clusters, as expected. Therefore, the three items pertaining to each dimension were averaged to form a total score of *perceived pervasiveness of moral evaluations* and *perceived pervasiveness of competence evaluations*, respectively.

We then assessed *anticipated ingroup respect* with a measure adapted from Pagliaro et al. (2011). Participants indicated on five bipolar scales how they anticipated *other members of their group* to react if they were to behave either in an immoral or in an incompetent way (“I think that they would: Exclude me-Include me; Reject me-Accept me; Shun me-Welcome me; Avoid me-Approach me; Ignore me-Appreciate me”). This measure was completed twice: First participants judged a situation in which they were seen as lacking in morality (α = 0.83) and then participants judged the implications of being seen as lacking in competence (α = 0.89). Although we did not counterbalance for order, it was made clear to participants from the start that they would have to answer these questions with respect to both dimensions, and the two sets of questions were printed on the same page.

### Results and Discussion

#### Perceived Pervasiveness of Morality and Competence

A repeated-measures Analysis of Variance (ANOVA) was performed on the perceived pervasiveness of morality and competence judgments. As predicted, moral judgments (*M* = 6.37; *SD* = 1.58) were considered significantly more pervasive than competence judgments (*M* = 5.97; *SD* = 1.73), *F*(1,125) = 4.86, *p* = 0.029, $\eta_{p}^{2}$ = 0.04.

#### Anticipated Ingroup Respect

An ANOVA on anticipated ingroup respect, with dimension (morality vs. competence) as a within participants factor revealed that participants anticipated receiving less ingroup respect when they imagined behaving in an immoral (*M* = 3.06; *SD* = 1.19) rather than in an incompetent way (*M* = 3.98; *SD* = 1.24), *F*(1,125) = 73.41, *p* < 0.001, $\eta_{p}^{2}$ = 0.37. This corroborates our reasoning that a negative evaluation stemming from the ingroup is seen as more consequential when it is based on moral (vs. competence) judgments.

## Study 2

Study 2 aimed to extend the evidence obtained in Study 1 and previous work in which judgments of dishonest behavior were seen as indicative of the broader dimension of communion (Ybarra et al., 2012). We did this: (a) by comparing moral judgments to competence and sociability judgments and (b) by including a broader range of measures to assess specific implications of the tendency to consider moral judgments to be more pervasive. In addition to assessing perceived pervasiveness of different types of judgments over time, we asked participants to indicate the perceived importance and centrality of such judgments for the self, in the eyes of others. We also asked whether participants saw such judgments as indicative of the true nature of a person, as predictive of future behavior, and as requiring effort to repair. Finally we asked participants to indicate the extent to which they thought that failing to meet moral vs. competence standards would be consequential for the likelihood of receiving intragroup respect. We expected that moral judgments would be seen as more pervasive, and hence receive higher ratings on each of these social implications of pervasiveness, than judgments of competence or sociability.

### Method

#### Participants

A total of 299 undergraduates (153 women; mean age = 24.04; *SD* = 5.69) were recruited on the university campus and anonymously answered a paper-and-pencil questionnaire. After each session participants were thanked and fully debriefed.

#### Procedure

Participants considered a list of nine judgments indicative of morality (*honest, sincere, trustworthy*), competence (*competent, intelligent, skillful*) and sociability (*friendly, warmth, kind*), adapted from Leach et al. (2007). For each of these nine judgments participants were asked to indicate (from 1 = *not at all*; to 9 = *completely*) to what extent (1) they considered it important to possess this characteristic for themselves (α: *Morality* = 0.73; *Competence* = 0.85; *Sociability* = 0.82); (2) they considered it important that others would see them as having this characteristic (α: *Morality* = 0.78; *Competence* = 0.86; *Sociability* = 0.83); (3) they thought this evaluation would remain stable over time (α: *Morality* = 0.69; *Competence* = 0.75; *Sociability* = 0.80); (4) they thought this characteristic was indicative of a person’s true nature (α: *Morality* = 0.73; *Competence* = 0.74; *Sociability* = 0.78), and (5) they thought of this characteristic as predictive of future behavior (α: *Morality* = 0.78; *Competence* = 0.73; *Sociability* = 0.83).

Then, participants considered what would happen if someone were to be seen by the ingroup as behaving in an immoral way, and if someone were to be seen by the ingroup as behaving in an incompetent way. Participants indicated in each of eight items the amount of effort that would be required to repair this type of negative evaluation stemming from the ingroup (1 = *Not at all*; 9 = *Very much*). Sample items are: To what extent would it be difficult: *To justify one’s behavior to other ingroup members; to try to repair the consequences of this behavior; to regain trust in the eyes of others* (α: *Morality* = 0.84; *Competence* = 0.68). The order in which moral and competence judgments were rated was counterbalanced, and did not influence the results.

Finally, participants indicated to what extent they considered a failure to meet the group’s standards of morality (α = 0.90) or competence (α = 0.86) to be socially consequential (*anticipated ingroup respect)* as in Study 1.

### Results

#### Mean Differences

For each variable, a repeated measures ANOVA was performed, with dimension (Morality vs. Competence vs. Sociability) as a within participants factor (see **Table 1** for relevant statistics). Differences in the degrees of freedom are due to missing values. In line with our general prediction, moral judgments were perceived as more pervasive and consequential than either competence or sociability judgments: Moral characteristics were perceived as more pervasive over time, as more important both for one’s view of oneself and for one’s perceived image in the eyes of others, as more indicative of the true nature of a person, and as more predictive of future behavior. These results also specify that morality judgments are perceived as more pervasive and all-encompassing than sociability judgments. Finally, participants indicated that more effort would be required to repair one’s image in the group in the moral domain compared to the competence domain.

**Table 1:** Means (and standard deviations) for morality, competence and sociability information and statistics of repeated measures ANOVAs (Study 2).

Target variable	Morality	Competence	Sociability	*F*, *p*, and $\eta_{p}^{2}$ values
Perceived pervasiveness over time	6.24^a^ (1.91)	5.86^b^ (2.01)	5.19^c^ (2.06)	*F*(2,298) = 28.86, *p* < 0.001; $\eta_{p}^{2}$ = 0.09
	
	Pairwise comparisons: M vs. C: *p* = 0.03; M vs. S: *p <* 0.001; C vs. S: *p <* 0.001			
	
Importance and centrality for the self	7.39^a^ (1.04)	7.21^b^ (1.33)	6.76^c^ (1.51)	*F*(2,298) = 87.93, *p* < 0.001; $\eta_{p}^{2}$ = 0.23
	
	Pairwise comparisons: M vs. C: *p <* 0.001; M vs. S: *p <* 0.001; C vs. S: *p <* 0.001			
	
Importance and centrality in the eyes of others	7.76^a^ (1.19)	7.09^b^ (1.45)	6.64^c^ (1.64)	*F*(2,298) = 83.60, *p* < 0.001; $\eta_{p}^{2}$ = 0.22
	
	Pairwise comparisons: M vs. C: *p <* 0.001; M vs. S: *p <* 0.001; C vs. S: *p <* 0.001			
	
Indicative of true nature	7.18^a^ (1.64)	5.29^b^ (1.88)	5.51^b^ (2.04)	*F*(2,298) = 106.67, *p* < 0.001; $\eta_{p}^{2}$ = 0.26
	
	Pairwise comparisons: M vs. C: *p <* 0.001; M vs. S: *p <* 0.001; C vs. S: *p* = 0.37			
	
Predictive of future behavior	7.05^a^ (1.63)	5.57^b^ (1.72)	5.53^b^ (2.01)	*F*(2,297) = 89.78, *p* < 0.001; $\eta_{p}^{2}$ = 0.23
	
	Pairwise comparisons: M vs. C: *p <* 0.001; M vs. S: *p <* 0.001; C vs. S: *p* = 0.26			
	
Requiring effort to repair	5.64^a^ (1.37)	4.32^b^ (1.14)	–	*F*(1,298) = 242.44, *p* < 0.001; $\eta_{p}^{2}$ = 0.45
Anticipated ingroup respect	3.49^a^ (1.29)	4.52^b^ (1.32)	–	*F*(1,295) = 172.09, *p* < 0.001; $\eta_{p}^{2}$ = 0.37


*Different superscripts in raw indicate a significant difference at least at *p* < 0.05. Pairwise comparisons were performed with Bonferroni tests. For the last two variables the comparison was between morality and competence information.*

#### Correlation Analyses

To examine how perceptions of pervasiveness relate to the perceived social implications of morality judgments, we correlated perceived pervasiveness of morality over time with the other variables considered in this study. These analyses provided support for our reasoning. That is, the perceived pervasiveness of morality over time related positively to the perceived importance of morality for self-views (*r* = 0.24, *p* < 0.001), to one’s perceived image in the eyes of others (*r* = 0.19, *p* = 0.01), to the belief that morality is indicative of the true nature of a person (*r* = 0.29, *p* < 0.001), and to the belief that it is predictive of future behavior (*r* = 0.20, *p* < 0.001). No reliable correlation emerged between perceived pervasiveness and the perceived effort required to restore one’s image (*r* = -0.09, *p* = 0.13)^1^.

## Study 3

Study 3 aimed to test whether participants’ inclination to explain and justify their behavior would be enhanced when self-relevant others (ingroup members) questioned their morality (vs. competence). We proposed that participants would be more inclined to justify and explain their behavior—as a way to restore their image in the eyes of others—when it was evaluated as immoral (rather than moral) by the ingroup. We argued that this effect would be mediated by the perceived pervasiveness of moral judgments. We predicted no comparable effects to emerge for negative competence evaluations.

### Method

#### Design and Participants

The design of the study was a 2 (*Dimension:* Morality vs. Competence) × 2 (*Evaluation of Behavior:* Positive vs. Negative) between participants factorial design. A total of 156 undergraduates took part in this study (98 women; mean age = 24.73; *SD* = 4.32). After each session participants were thanked and fully debriefed.

#### Procedure

##### Behavioral descriptions

Participants were asked to think of two situations where their behavior was evaluated by members of their ingroup (i.e., self-relevant others). To control for the content of the behavior, we selected behaviors that would seem realistic to participants and were sufficiently ambiguous to make it plausible that others observing this behavior might have different interpretations as to what this behavior indicated about their morality. Specifically, they were asked to consider the situation in which they had conveyed to close friends (a) that they often break speed limits when driving their car, but slow down in proximity of speed control checks (scenario 1); (b) that they benefited of an irregular recommendation in order to get a job (scenario 2) (for a similar procedure but with different scenarios, see Ybarra et al., 2012; Study 2).

##### Manipulation of moral/competence evaluation

Participants were asked to imagine that they overheard their close friends talking about the behavior they had described and evaluating it positively or negatively, along the moral or the competence dimension (according to condition). In the *positive moral* condition, participants read that their close friends had described their behaviors as moral and honest— and this was illustrated with a concrete example. In the *positive competence* condition participants read that their behavior had been evaluated as intelligent and smart—here too an example was cited. In the *negative moral* condition, participants read that their close friends had evaluated their behavior as immoral and dishonest. Finally, in the *negative competent* condition, participants read that their behavior was evaluated as stupid and incompetent. We took care to choose ‘everyday’ behavioral examples (driving behavior, applying for a job) and kept these constant across conditions. The behaviors that were chosen were somewhat ambiguous, so that different judgments could be attached to the same behavior, depending on which aspect was focused on. The face validity of these evaluations was enhanced in each case by providing a plausible explanation of the situation in line with the judgments made. For instance slowing down in proximity of speed control checks was depicted, according to condition, either as an immoral behavior since it does infringe others’ right, or as a stupid behavior, since it endangers the driver. The emphasis thus was on the social evaluations conveyed, not on the concrete behavior that had prompted this evaluation. These manipulations were checked by asking participants to indicate how they thought their friends had evaluated their behaviors in terms of competence (from 1 = *very stupid;* to 9 = *very smart*) and morality (from 1 = *very immoral;* to 9 = *very immoral*).

Participants completed the same measures as in Study 1 to assess *perceived pervasiveness of morality* (α = 0.77) and of *competence* (α = 0.79). A PCA again revealed a 2-factor solution accounting for 70.13% of the variance, with the six items loading on the intended factors.

##### Inclination to restore social image

Participants indicated the extent to which they would be inclined to justify and explain their behavior to other ingroup members as a way to influence their social image (to what extent would you be willing to: “explain your own behavior to other ingroup members”; “invest time trying to justify your behavior to your ingroup members”; *r* = 0.56, *p* < 0.001; from 1 = *not at all;* 9 = *very much*). Answers to these items were averaged to indicate the inclination to restore one’s image in the group.

To assess *anticipated ingroup respect*, participants indicated how they anticipated *other members of their group* to react in the situation they had just considered (i.e., in which their behavior was characterized by ingroup representatives as indicating a lack of morality vs. competence, depending on experimental condition; α = 0.94).

### Results and Discussion

#### Manipulation Checks

We conducted two separate one-way ANOVAs for each manipulation check testing the effect of *Evaluation of Behavior* (Positive vs. Negative) as a between participants factor. As regards morality, the analysis confirmed that participants reported that other ingroup members had evaluated their behaviors as less moral in the negative (*M* = 3.75; *SD* = 1.18) than in the positive moral condition (*M* = 7.85; *SD* = 1.27), *F*(1,74) = 451.519, *p* < 0.001, $\eta_{p}^{2}$ = 0.43. Similarly, as regards competence, participants reported that other ingroup members had evaluated their behaviors as significantly less competent in the negative (*M* = 5.60; *SD* = 2.44) than in the positive competence condition (*M* = 6.80; *SD* = 1.96), *F*(1,78) = 5.91, *p* = 0.017, $\eta_{p}^{2}$ = 0.07). This indicates that our manipulations had the intended effect on participants’ perceptions.

#### Perceived Pervasiveness of Morality and Competence

A repeated-measures ANOVA only revealed a significant effect of dimension, *F*(1,155) = 12.08, *p* = 0.001, $\eta_{p}^{2}$ = 0.07. In line with predictions, morality information (*M* = 6.49; *SD* = 1.96) was considered as more pervasive than competence information (*M* = 5.77; *SD* = 2.04), irrespective of its valence.

#### Anticipated Ingroup Respect

A 2 (*Dimension:* Morality vs. Competence) × 2 (*Evaluation of Behavior:* Positive vs. Negative) between participants ANOVA revealed no main effect of Dimension, *F*(1,151) = 0.047, *p* = 0.829, but a significant effect of Evaluation of Behavior, *F*(1,151) = 213.79, *p* < 0.001, $\eta_{p}^{2}$ = 0.59. Importantly, this effect was qualified by a two-way interaction, *F*(1,151) = 10.82, *p* = 0.001, $\eta_{p}^{2}$ = 0.07. In line with predictions, inspection of simple effects confirmed that in the *Negative* Evaluation condition participants anticipated receiving *less* respect when the evaluation of their behavior implied they were lacking in morality (*M* = 2.34; *SD* = 1.04) rather than competence [*M* = 2.91; *SD* = 1.05; *F*(1,155) = 4.57, *p* < 0.05, $\eta_{p}^{2}$ = 0.03]. In the *Positive* Evaluation condition participants anticipated receiving *more* respect when the evaluation highlighted the morality of their behavior (*M* = 5.69; *SD* = 1.22) rather than the competence of their behavior [*M* = 5.03; *SD* = 1.29; *F*(1,155) = 6.36, *p* = 0.01, $\eta_{p}^{2}$ = 0.04]. Thus, in line with our reasoning and prior results, participants anticipated the social implications of evaluative judgments from other ingroup members to be more extreme when these referred to their morality compared to their competence.

#### Inclination to Restore Social Image

A similar 2 × 2 between participants ANOVA revealed a significant effect of Evaluation of Behavior, *F*(1,152) = 9.33, *p* = 0.003, $\eta_{p}^{2}$ = 0.06, and a significant effect of Dimension, *F*(1,152) = 6.45, *p* = 0.012, $\eta_{p}^{2}$ = 0.04, but both main effects were qualified by the predicted two-way interaction, *F*(1,152) = 8.39, *p* = 0.004, $\eta_{p}^{2}$ = 0.052.

In line with our predictions, inspection of simple effects revealed that the effect of Evaluation of Behavior was only significant in the Morality condition: Participants expressed a stronger intention to justify and explain their behaviors to other ingroup members when these had evaluated them as immoral (*M* = 7.42; *SD* = 1.41) rather than moral (*M* = 5.70; *SD* = 2.21), *F*(1,152) = 14.39, *p* < 0.001, $\eta_{p}^{2}$ = 0.09. By contrast, no differences emerged in the Competence condition (Positive: *M* = 5.65; *SD* = 2.13; Negative: *M* = 5.54; *SD* = 1.96), *F*(1,152) = 0.065, *p* = 0.779. As a result, and again in line with predictions, the difference between Morality and Competence evaluations was significant only in the Negative condition, with participants expressing more willingness to invest in restoring their social image when other ingroup members had evaluated them as immoral rather than incompetent, *F*(1,152) = 17.24, *p* < 0.001, $\eta_{p}^{2}$ = 0.10.

#### Mediation Analyses

We tested for the mediating effects of perceived pervasiveness of moral judgments on participants’ justifications. Because the effect to be mediated is a Dimension by Evaluation of Behavior interaction, we tested for mediated moderation (Muller et al., 2005). As reported above, the interaction effect on justifications and explanations was significant (β = 0.21, *p* = 0.009). Also, the two-way interaction predicted perceived pervasiveness of morality, β = 0.24, *p* = 0.003, and pervasiveness of morality predicted justifications, β = 0.26, *p* = 0.001. When this interaction and the perceived pervasiveness of morality were entered simultaneously as predictors, the effect of pervasiveness of morality remained significant (β = 0.23, *p* = 0.005), while the relationship between the interaction and justifications was no longer reliable (β = 0.15, *p* = 0.06). Bootstrapping with 5,000 resamples and 95% confidence intervals indicated that there was a significant indirect effect of our manipulations on willingness to invest in restoring one’s moral image, through perceived pervasiveness of morality (95% CI: LL = 0.0092; UL = 0.2902).

### Discussion

Study 3 expanded our knowledge about the processes that underlie the effects of moral judgments on individual behavior. Results suggest that moral judgments impact on the behavioral motivation of individuals to the extent that their reputation and inclusion are at stake. Additionally, Study 3 showed that the perceived pervasiveness of moral judgments has a unique role in predicting the tendency to invest in restoring one’s social image, as pervasiveness of competence judgments was not related to restoration efforts.

## Study 4

Study 4 aimed to examine willingness to invest in restoring one’s social image with a more elaborate measure. Additionally, we aimed to elaborate on the underlying mechanisms that explain the effect of moral evaluations on the willingness to invest in restoring one’s social image. Specifically, we examined whether a negative morality evaluation elicits threat and is perceived as difficult to cope with and whether this, in turn, enhances people’s willingness to invest in restoring their social image.

### Method

#### Design and Participants

Morality-based Evaluation of Behavior (*Moral vs. Immoral*) was manipulated between participants. A total of 321 undergraduates took part in this study (166 women; mean age = 23.76; *SD* = 5.91). After completing the questionnaire participants were thanked and fully debriefed.

#### Procedure

The procedure was identical to that of Study 3, except that now only morality evaluations were manipulated. Also, the dependent variables were extended. Specifically, after reading the behavioral descriptions and the judgments made by the self-relevant others, participants indicated the extent to which the evaluation they had received would elicit *uncertainty, anxiety, stress, fear*, and *insecurity* (from 1 = *not at all* to 9 = *very much*). These responses were averaged to create a single *threat* index (α = 0.87).

Participants then completed a measure of *perceived coping abilities*. They were asked to think about the situation described, and answer four questions assessing their perceived ability to manage the situation (e.g., “I’m certainly able to manage this situation”; “This situation is very demanding” (recoded); α = 0.87). Given the theoretical closeness between measures of *threat* and *perceived coping abilities*, we performed a PCA with Oblimin rotation, which confirmed a 2-factor solution accounting for 63% of the total variance, with the items clustered as intended on two correlated factors (*r* = -0.50, *p* < 0.001).

*Anticipated ingroup respect* was assessed as in the prior studies (α = 0.91). Finally, we assessed *inclination to restore one’s social image* with a more elaborate measure than in the previous studies. Participants indicated the extent to which they were inclined to make various kinds of efforts that might help restore their image in the eyes of ingroup members (“explain your behavior to other ingroup members”; “invest time in trying to justify your behavior to other ingroup members”; “make an effort to change your image in the eyes of other ingroup members”; “actively engage in attempts to contradict the image that your friends have of you”; “make an effort to justify your behavior to other ingroup members”). Answers to these questions (1 = *not at all;* to 9 = *very much*) were averaged to indicate participants’ inclination to invest in restoring their social image (α = 0.80).

### Results

For each relevant variable, a one-way ANOVA was performed, with Morality-based Evaluation of Behavior (Moral vs. Immoral) varying between participants. Differences in the degrees of freedom are due to missing values.

#### Manipulation Check

The ANOVA confirmed that participants in the *immoral* condition indicated that other ingroup members had evaluated their behavior as less moral (*M* = 6.16; *SD* = 2.78) than participants in the *moral* condition (*M* = 6.69; *SD* = 2.28), *F*(1,318) = 4.93, *p* = 0.027.

#### Anticipated Ingroup Respect

Participants anticipated to receive less respect when their behavior was evaluated as immoral (*M* = 2.93; *SD* = 1.49) than when it was evaluated as moral (*M* = 4.84; *SD* = 1.49), *F*(1,322) = 155.14, *p* < 0.001, $\eta_{p}^{2}$ = 0.33.

#### Inclination to Restore Social Image

The ANOVA showed that, as anticipated, participants were more willing to make an effort to restore their image when their moral behavior had been evaluated negatively (*M* = 5.49; *SD* = 1.98) rather than positively (*M* = 4.96; *SD* = 1.84), *F*(1,318) = 5.93, *p* = 0.015, $\eta_{p}^{2}$ = 0.02.

#### Threat and Coping Ability

The ANOVA showed that participants indicated higher levels of threat in response to a negative moral evaluation (*M* = 3.60; *SD* = 2.04) than after a positive moral evaluation (*M* = 2.94; *SD* = 1.79), *F*(1,324) = 9.83, *p* = 0.002, $\eta_{p}^{2}$ = 0.03. At the same time, participants reported lower perceived coping abilities in the negative evaluation condition (*M* = 5.48; *SD* = 1.71) than in the positive condition (*M* = 6.17; *SD* = 1.81), *F*(1,324) = 12.39, *p* < 0.001, $\eta_{p}^{2}$ = 0.04. This confirms that receiving a negative moral evaluation from other ingroup members is perceived as highly stressful and difficult to cope with, as predicted.

#### Mediation Analyses

**FIGURE 1:**
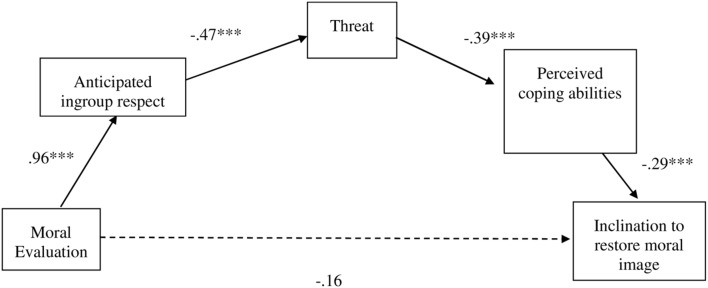
**Model with multiple sequential mediators (Study 4).** PROCESS Model number 6; unstandardized regression coefficients are presented in the figure; ^∗∗∗^*p* < 0.001.

We tested a sequential multiple mediator model (see **Figure 1**). According to our rationale, individuals’ attempts to restore their moral image should be driven by concerns related to their acceptance and inclusion in the group. Therefore, we tested whether moral evaluations (positive vs. negative) impact on anticipated intragroup respect, which in turn elicits more threat and lowers perceived coping abilities—and these, in turn, enhance the inclination to invest in restoring one’s image in the group. We followed the procedure described by Preacher and Hayes (2004) for estimating direct and indirect effects with multiple potential mediators (PROCESS model number 6). The bootstrapping procedure with 1,000 resamples indicated that the indirect effect reflecting the causal chain we hypothesized was significant (*b* = -0.05; 95% CI: LL = -0.1058; UL = -0.0293). In comparison, the direct effect in the full model was not significant (95% CI: LL = -0.3945; UL = 0.0715), showing that our proposed mediators accounted for the effect of moral evaluations on individuals’ inclination to restore their image in the group^2^.

## General Discussion

This research examined the relation between the importance people attach to moral judgments of the individual *self*, the way they think this relates to the respect they receive from other ingroup members,^3^ and attempts to restore one’s moral image. Results from four studies show that people perceive moral evaluations as more *pervasive* than evaluations of their competence or sociability. As a result, they think negative moral judgments of the self are likely to be more harmful for the respect they receive from others who are important to them. This, in turn, motivates attempts to restore a moral image to a greater extent than attempts to restore a competent reputation.

These results offer support for the notion that moral judgments have a social regulatory function (Ellemers and van den Bos, 2012), but it expands what has been already shown in the literature (e.g., Ybarra et al., 2012), by addressing the perceived pervasiveness of moral judgments as a key mechanism explaining the impact of these judgments on impression management processes. We also connect to prior research on behavioral regulation in groups (Ellemers et al., 2013), by demonstrating the importance of respect and inclusion by self-relevant others in this process.

By showing the importance of morality for the evaluation of the individual self we do not want to deny the role that other evaluative dimensions such as competence may play. Nevertheless, despite some studies showing that contextual features (e.g., the goal of the evaluation) may moderate the salience of specific evaluative dimensions (e.g., Brambilla et al., 2012), there is by now considerable evidence showing that even in truly competence-driven contexts (such as in task teams or work organizations; see for instance Ellemers et al., 2011; Van Prooijen and Ellemers, 2015) morality emerges as the strongest guide for individuals’ behavior. This is not to say, of course, that competence is irrelevant: but, when moral concerns are made explicit, people primarily strive to be considered moral (even if this means to be considered as less competent; see Ellemers et al., 2008; Pagliaro et al., 2011).

### Implications

In this paper, we have revealed that people care about moral judgments of the self and tend to behave in ways that protect and restore their moral image in the eyes of others because they see these as pervasive predictors of respect and inclusion (see also Ellemers and Jetten, 2013). These insights may inform organizational practice. The present results suggest that evaluating employees in moral terms may be more effective in guiding and directing their behavior than evaluating them in purely competence terms, producing, for instance, greater compliance with organizational policies. However, this is more likely to be effective if those evaluating the moral behavior of employees are seen as ingroup members rather than as representatives of the outgroup (Ellemers, 2001; Ellemers et al., 2004; Haslam and Ellemers, 2005). While moral evaluations and raising inclusion concerns may seem to offer a highly effective way to influence the behavior of individuals, such use of moral judgments raises at least two additional concerns. First, those who feel their moral image is beyond repair may become disaffected as a way to cope with the threat of moral exclusion. That is, initial messages communicating disrespect from the group may motivate individuals to make an effort to exert themselves on behalf of the group to show they are worthy of group membership. However, continued exposure to disrespect from other group members makes people want to leave the group, as they give priority to saving their own image rather than continuing to seek inclusion in a group that does not value them (Sleebos et al., 2006a,b). So while our studies reveal that people will attempt to restore their moral image, when threatened, they also clarify that people do so partly because they recognize that this is a hard thing to do, especially if initial questions remain unaddressed.

A second issue is that, if moral evaluations are perceived as threatening and hard to cope with, they raise defensive responses and may thus not constitute the best way to achieve behavioral *change*, for instance through engagement with new policies or additional guidelines. This, however, may depend on how moral evaluations are *conveyed*. Indeed, recent research revealed that people were willing to adapt to moral *ideals* they might try to achieve (Does et al., 2011). However, when the same goal was communicated to them as a moral *obligation* they needed to fulfill, this raised a physiological threat response, and made people less willing to support actions aiming to achieve this (Does et al., 2012). This suggests that people may be more open to consider and adapt their behavior if behavioral goals are communicated as *positive* moral actions they might adopt, rather than in terms of negative moral actions they should avoid.

### Limitations and Future Directions

We orthogonally manipulated competence vs. morality judgments of the self, in a way that may be seen as artificial. Indeed, in more naturally occurring situations behavioral approval or disapproval tends to be conveyed in a more general sense, and people tend to assume that overall evaluative judgments extend to different evaluative dimensions. Future research might examine whether and how people make such inferences, and whether this is the same for ingroup and outgroup judgments. The perceived pervasiveness of moral judgments compared to competence judgments is likely to be relevant here. For instance, we would expect that information about someone’s morality is also seen to inform competence judgments, while competence judgments have less of a spill-over effect.

Another potential limitation is that we described specific scenarios to elicit participants’ reactions to different types of situations. Even though we note that the results of different studies revealed consistent effects with different methodologies, future research might expose participants to a more immersive situation, in which they actually receive other people’s judgments of the behavior they displayed. At the same time, we note that the current methodology is of interest in its own right, as it represents a type of situation that also occurs in real life. That is, our aim to examine meta-perceptions of the self implies that—by definition—we are interested in how people *imagine* they will be judged by others, and adapt their behavior depending on the approval or disapproval they anticipate. Indeed, the scenarios we used in some of our studies capture this aspect quite accurately, as moral criticism is rarely voiced openly in social interactions. Instead, people mostly draw their own conclusions about the way they are judged by others by interpreting indirect hints, through hearsay, or by overhearing gossip. Thus, despite the fact that the methodology we used only captures a specific class of situations, and despite the procedure in which participants had to *anticipate* what they would feel like when being evaluated in this way, this approach remains ecologically valid, as it represents a type of situation that also occurs in real life.

A related issue is the fact that in this series of studies we adopted self-report measures and assessed behavioral intentions. Future studies might also include behavioral outcomes of these processes. Empirical data available so far, give us no reason to believe that this will yield different outcomes. For instance, recent research on the role of morality in impression formation (Iachini et al., 2015) used an immersive virtual reality paradigm (IVR). Results from this research show that actual behavior—in this case their tendency to physically approach or avoid others—is consistent with results of prior work, relying on self-report measures about approach vs. avoidance tendencies (e.g., Brambilla et al., 2012, 2013; Pagliaro et al., 2013). This also raises confidence in the fact that the current self-report data are likely to translate into actual behavioral efforts.

## Conclusion

Moral concerns are seen as providing an important guideline in individual decision making and behavior. At the same time, we know very little about how moral judgments affect self-views and efforts toward moral impression management within social groups. The present research supports our analysis that people are motivated to restore their positive moral image when this is called into question by a negative evaluation from relevant others. We further demonstrated that people are inclined to invest this type of effort because they see this as a way to regain social respect. This has important implications for current theories on moral psychology and social identity, and opens up exciting new perspectives for applying these theories in practice.

## Author Contributions

SP, NE, and MB developed the present research, and contributed to writing the paper. SP and CD collected and analyzed the data.

## Conflict of Interest Statement

The authors declare that the research was conducted in the absence of any commercial or financial relationships that could be construed as a potential conflict of interest.

## Footnotes

1 To control for the effects of perceived pervasiveness of competence and sociability, we also calculated partial correlations, correcting for the variance associated with these other dimensions of judgment. These analyses provide additional support for our reasoning that moral judgments have unique effects, that cannot be explained from their overlap with competence or sociability judgments. Specifically, also after controlling for competence and sociability ratings, we found that the perceived pervasiveness of morality over time related positively to the perceived importance of morality for self-views (*r* = 0.21, *p* < 0.001), to one’s perceived image in the eyes of others (*r* = 0.15, *p* = 0.01), to the belief that morality is indicative of the true nature of a person (*r* = 0.32, *p* < 0.001), and to the belief that it is predictive of future behavior (*r* = 0.21, *p* < 0.001).

2 We checked whether an alternative causal order showed a statistically better fit to the data. Specifically, we tested an alternative model, to examine whether threat raises lack of respect, and this results in poor coping. This reveals a significant relation, but the indirect effect is less strong (*b* = -0.006; CI 95%: LLCI: -0.0163; ULCI: -0.0022) than the indirect effect in the model we have hypothesized and tested. The threat and coping literature (e.g., Lazarus and Folkman, 1984) distinguishes between primary appraisals and secondary appraisals of taxing situations. The primary appraisal refers to how threatening or demanding the situation is, the secondary appraisal refers to the individual’s perceived ability to deal with these demands. Thus the causal order we tested thus best fits the data and best reflects the theoretical rationale connecting these constructs.

3 Further support for this reasoning stems from the results of another study (Pagliaro et al., unpublished) in which participants were instructed to indicate how they would respond to evaluations of their behavior provided by others who were not relevant to the self (i.e., an outgroup). In this case, effects of moral evaluations were not different from effects of competence evaluations.
